# *Toxoplasma gondii* Infection and Proinflammatory Cytokine Level Assessment in Patients With Myocardial Infarction in Iraq: A Case–Control Study

**DOI:** 10.1155/cjid/2553949

**Published:** 2026-03-02

**Authors:** Al-Abbas Fadhil Jasim, Amal Khudair Khalaf

**Affiliations:** ^1^ Department of Microbiology, College of Medicine, University of Thi-Qar/Iraq, Nasiriyah, Iraq, medcol.mw

**Keywords:** cardiovascular, ELIA, IFN-γ, inflammation, TGF, toxoplasmosis

## Abstract

**Background:**

*Toxoplasma gondii* infections have been implicated in cardiac complications in both humans and animals, including myocarditis and pericarditis. This study surveyed the frequency of *T. gondii* antibodies and investigated the serum concentrations and gene expression profiles of interferon‐gamma (IFN‐γ) and transforming growth factor‐beta (TGF‐β) in myocardial infarction (MI) patients in Iraq.

**Methods:**

This study was conducted on MI (case) and non‐MI healthy (control) individuals (200 participants/each group) in Baghdad, Iraq. The frequency of IgG and IgM anti‐*Toxoplasma* antibodies and the serum and expression level of IFN‐γ and TGF‐β were assessed by serological and real‐time PCR.

**Results:**

The frequency of anti‐*Toxoplasma* IgG and IgM antibodies in the patient group was reported to be 55.5% and 4.5%, respectively, while in the non‐MI group, this rate was observed to be 42.9% and 2.5%. In addition, both serum levels and gene expression levels of IFN‐γ and TGF‐β were meaningfully higher in the MI group compared to the non‐MI control group (*p* ≤ 0.001).

**Conclusion:**

The present study revealed that individuals with MI exhibited significantly higher concentrations of anti–*T. gondii* antibodies compared to non‐MI. The data further suggest that IFN‐γ and TGF‐β serve as critical biomarkers connected in the pathogenesis of MI. Our findings propose that *T. gondii* may contribute to the development of MI via immune‐mediated inflammatory pathways. This observation indicates potential molecular similarities between the pathological elements of MI and the *T. gondii* pathogen. Moreover, the elevated expression of IFN‐γ and TGF‐β may offer a respected context for investigating the involvement of *T. gondii* in MI pathophysiology.

## 1. Introduction

According to authoritative sources, it is significant to note that around 70% of the universal population are serum positive for *Toxoplasma gondii* parasite [1]. The primary modes of transmission involve the ingestion of the parasite in the form of cysts present in raw/undercooked meat and water, fruits, and vegetables contaminated with parasite [2, 3]. While the majority of immunocompetent individuals endure no sign, those with compromised immune systems are at heightened risk for developing clinical problems linked with toxoplasmosis [4]. The disease has been accompanying to cardiac pathologies in both human and animal hosts. In humans, *T. gondii*–related cardiac involvement includes myocarditis, pericarditis accompanied by myocarditis, pericardial effusion, heart failure, and several arrhythmias [5].

Cell‐mediated immune response plays an important part in generating an immune response in the host to control infections caused by pathogens such as *T. gondii*. Proinflammatory cytokines, namely, IFN‐γ, TGF‐β, TNF‐α, IL‐12, IL‐1, and IL‐18, play vital roles in triggering and sustaining immune response and immunity in the host, as they lead to the inhibition of *Toxoplasma* parasite growth [6]. IFN‐γ is specifically designated as the major cytokine in relation to resistance mechanisms in the host infected by *T*. *gondii* in the course of an immune response in the early and chronic phases of infection, as in toxoplasmic encephalomyelitis and myocarditis [6]. It has also been indicated in the literature that TGF‐β plays an important role in generating an immune response in the host to overcome infections caused by *T. gondii* [7]. The important functions of TGF‐β in relation to immune response and immunity in the host have been elucidated in the literature. Specifically, the main roles of TGF‐β in relation to immunity in the host in infections caused by *T. gondii* have also been indicated in the literature, thus providing more insight in relation to the role of TGF‐β and infections caused by pathogenic parasites such as *Toxoplasma* [8].

The current literature substantiates this idea and introduces the idea that there is a key role played by immune activation related to the current diseases affecting the heart [9]. Previous clinical studies have noted and have been able to show leukocyte infiltration to the heart tissue, immune cell activation, and a higher concentration of proinflammatory cytokines such as TNF‐α, IL‐1β, and IL‐6 from the plasma and heart tissue from the hearts of individuals undergoing a heart disease process [10]. Previous proteomic profiles have noted and been able to show a process where exposure to IFN‐γ causes mitochondrial dysfunction and changes to mitochondrial outer membrane potential as well as a disturbance to fatty acid metabolism and features related to heart tissue necrosis and cell death itself [11]. Another problem noted in heart tissue is the development and process known as cardiac fibrosis, where there is a process involving the differentiation and overexpression of myofibroblasts and the overexpression and deposition of matrix molecules themselves [12]. A major factor noted as leading to heart disease is TGF‐β; this factor is related to heart disease and to heart fibrosis, apoptosis involving heart tissue, TGF‐β played a vital role in cardiac fibrosis, cardiomyocyte apoptosis, ventricular hypertrophy development, and ultimately, in the progression to heart failure. Levels of TGF‐β are increased under circumstances of myocardial infarction (MI), thereby augmenting tissue injury [13].

In accordance with the World Health Organization (WHO), the prevalence of heart‐related diseases signifies the major mortality rate in the world, owing to 18 million deaths annually, comprising an estimated 32% of global deaths [14]. Understandably, the seroepidemiology of toxoplasmosis in relation to patients with heart‐related diseases has not yet received due attention in the available literature [14]. This is principally because cardiovascular manifestations of *T. gondii* infection are not usually expressed. Specifically, this is because signs and symptoms are not usually expressed in the presence of neuropsychiatric worsening. This study surveyed the frequency of *T. gondii* antibodies and investigated the serum concentrations and gene expression profiles of IFN‐γ and TGF‐β in MI patients in Iraq.

## 2. Materials and Methods

### 2.1. Ethics

Recruits for the participants were carried out in strict observance of the guidelines laid down in the Declaration of Helsinki, 1964. Thus, informed consent for all participants, including their respective legal guardians for illiterate participants who were sick literates, was obtained. The ethical examination for this research received formal approval by the Institutional Review Board of the College of Medicine, University of Thi‐Qar, Iraq, with approval number 13835/2024.

### 2.2. Study Design and Participants

The total number of patients included was 200 diagnosed with MI, while the total number of controls was 200 from a healthy population without MI. The place of origin for this specific case–control study was conducted at the main cardiac centers located at Baghdad Governorate, Iraq. The period for which this specific case–control study was conducted was between September 2023 and October 2024. For diagnosing MI among specific groups of a case group during specific periods of time at specific places of origin, a thorough check‐up was conducted among together with specific tests conducted with the supervision of certified cardiologists. In contrast, for selecting controls under this specific case–control study, they were collected using nonprobability quota sampling methods. Exclusion criteria for controls include those people who had received systemic antibiotics during specific periods of time (e.g., cotrimoxazol, clindamycin, sulfadiazine, atovaquone, and azithromycin), together with those people who were immunocompromised (e.g., those people taking chemotherapy for malignancies and those people who have undergone an organ transplant together with those people diagnosed with Acquired Immunodeficiency Syndrome).

### 2.3. Questionnaire

Before the actual process of collecting blood samples, participants were instructed to first fill out a physical questionnaire that aimed to elicit certain demographic information, such as gender, age, and location of residence, among others.

### 2.4. Blood Collecting

Five milliliters of the sampled human blood were extracted through the sterile venipuncturing method, which used a hypodermic needle inserted through a disposable plastic syringe to penetrate the human veins, and the drawn human blood was then stored in a gel tube to coagulate for a period of an hour after the extraction procedure, after which the extracted human blood was subjected to centrifugation through the use of a centrifuging equipment operating through an angular velocity of 5000 revolutions for a period of 10 min, after which the human sera were equally parted for the immunologic test and chilled to a temperature of −20 °C.

### 2.5. Enzyme‐Linked Immunosorbent Assay (ELISA) for the Detection of Anti‐*Toxoplasma* Antibodies

The prevalence of anti–*T. gondii* antibodies, specifically IgG and IgM, was assessed using human *Toxoplasma*‐specific assays. This analysis was conducted employing the human *Toxoplasma* IgG and IgM ELISA kit (Elabscience, USA). These kits possess diagnostic sensitivities and specificities of 98.6% and 99.8%, respectively. All serum samples were tested following the manufacturer’s protocols. Laboratory procedures were designed to maintain a blinded approach, whereby the technician performing the assays was unaware of the clinical position of the participants. In cases where samples tested positive, referral to an infectious disease specialist was recommended.

### 2.6. Measurement of Cytokine Serum Levels via ELISA

The serum levels of IFN‐γ and TGF‐β were quantified utilizing commercial ELISA Kit (ELK Biotechnology, USA), following the protocols provided by the manufacturer. Subsequently, the levels of these cytokines were determined in picograms/milliliter (pg/mL) through the application of a standard curve and regression analysis, as delineated in the producer’s guidelines.

### 2.7. Assessment of Gene Expression via Quantitative Real‐Time PCR

Total RNA was isolated from PBMCs using a kit‐based process devised and sold by Qiagen, Germany, catalog number 74134. The concentration and quality of the purified RNA samples were checked and validated using a Nanodrop spectrophotometer from Biotek Epoch, USA. The purified RNA samples were converted to corresponding C‐DNA molecules through a kit‐based process devised and sold by Qiagen, Germany, catalog number 205311, according to manufacturer instructions and protocols. Quantitative polymerase chain reactions were carried out on C‐DNA samples using RT‐SybrGreen qPCR master mixes, catalog number 330513 from Qiagen, Germany, and upregulated target gene and β‐actin gene primers, as shown in Table 1, were used to execute the quantitative polymerase chain reactions as follows: A preliminary denaturation step was carried out, 40 amplification cycles were performed, final denaturation was done at 97 °C for 13 s, and annealing/extension was done at 63 °C and 30 s by the RT‐SybrGreen qPCR master mixes on a quantitative polymerase chain reaction machine, as shown in Table 2.

**TABLE 1:** Primers used in the real‐time PCR method.

Amplicon	Sequence (5′–3′)
IFN‐γ	F: ATTCGGTAACTGACTTGAATGTCC R: CTCTTCGACCTCGAAACAGC
TGF‐β	F: GCAAGTGGACATCAACGGGTT R: CGCACGCAGCAGTTCTTCTC
β‐actin	F: AAGCTCATTTCCTGGTATGACAACG R: TCTTCCTCTTGTGCTCTTGCTGG

**TABLE 2:** Seroprevalence of anti–*Toxoplasma gondii* IgG and IgM antibodies among patients diagnosed with myocardial infarction (MI) and non‐MI participants in Iraq.

Group	Participants	IgG antibody		IgM antibody	
		Positive no (%)	Negative no (%)	Positive no (%)	Negative no (%)
Non‐MI (control)	200	92 (42.5)	108 (57.5)	9 (4.5)	193 (95.5)
MI	200	111 (55.5)	89 (44.5)	4 (2.5)	196 (97.5)

### 2.8. Statistical Analysis

Statistical analysis of results was done utilizing SPSS software, i.e., Version 26.0. Chi‐square tests were used in order to check for any differences in the participants’ distributions between case and control groups, along with observing any associations between infection and factors of investigation. Additionally, any factor showing significant associations with the prevalence of *Toxoplasma* infection was further used for assessment of any potential associations with infection as “risk factors” utilizing “univariate logistic regression.” However, it was decided that any factor showing significant effects at a level of *p* ≤ 0.05 would pass as an established significance level. Moreover, in order to ascertain significant associations between the levels of IFN‐γ and TGF‐β in comparison with each of the studied groups, one way ANOVA along with Tukey’s test has been applied, respectively.

## 3. Results

### 3.1. Participants

The mean age of participants was 52.7 ± 8.12 years in the MI group and 53.4 ± 9.24 years in the non‐MI group. Males constituted a predominant proportion of the study population in both groups, with 150 males (75.0%) in the MI group and 144 males (72.0%) in the non‐MI group. About residential status, 59.0% in each group resided in urban areas, while the remainder were from rural regions.

### 3.2. Anti–*T. gondii* Antibodies’ Prevalence

The frequency of anti‐*Toxoplasma* IgG and IgM antibodies in the patient in the MI group was reported to be 55.5% and 4.5%, respectively, while in the non‐MI group, this rate was observed to be 42.9% and 2.5% (Table 1) with no significant difference.

### 3.3. The Related Risk Factors of Infection

Univariate analysis showed a strong statistical relationship between the presence of *T. gondii* antibodies and factors such as age (*p* ≤ 0.001), participant gender (*p* ≤ 0.001), and the habit of eating raw or undercooked meat (*p* ≤ 0.001) across both MI and non‐MI groups. In contrast, no meaningful link was detected between involvement in agricultural work or residential status and the frequency of *T. gondii* antibodies in either population (Tables 3 and 4). Further multivariate logistic regression indicated that individuals aged 30–40, those consuming raw or undercooked meat, and women had 2.14 (*p* ≤ 0.05), 2.26 (*p* ≤ 0.01), and 3.21 (*p* ≤ 0.01) times greater risk of toxoplasmosis, respectively (see Table 5).

**TABLE 3:** The seroprevalence of *Toxoplasma gondii* infection among patients diagnosed with myocardial infarction (MI) in Iraq considering demographic and risk factors utilizing chi‐square tests and univariate logistic regression analysis.

Variable	MI patients (*N* = 200) Anti‐*Toxoplasma* IgG antibody					
	Positive no (%)	Negative no (%)	^∗^ *p* value (chi‐square)	Odds ratio	95% CI	*p* value
*Age*						
< 30 yrs	2 (33.3)	4 (66.6)				
30–60 yrs	75 (52.8)	67 (47.2)	0.002^∗^	2.32	1.341–3.541	*p* ≤ 0.05^∗^
60 < yrs	34 (65.4)	18 (34.6)				

*Gender*						
Male	70 (46.6)	80 (53.4)		4.62	(1.821–8.321)	*p* ≤ 0.001^∗^
Female	41 (82.0)	9 (8.0)	0.000^∗^			

*Residence*						
Urban	66 (55.9)	52 (54.1)	0.671	1.28	0.834–1.934	0.364
Rural	45 (54.8)	37 (55.2)				

*Agriculture activity*						
No	71 (54.6)	59 (45.4)				
Yes	40 (57.1)	30 (42.9)	0.514	1.43	(0.916–2.892)	0.089

*Consumption of raw/uncooked meat*						
No	73 (47.1)	82 (52.9)				
Yes	38 (84.4)	7 (15.6)	0.000^∗^	3.577	(1.618–5.907)	*p* ≤ 0.001^∗^

^∗^ *p* value < 0.05 is a significant difference.

**TABLE 4:** The seroprevalence of *Toxoplasma gondii* infection among healthy control individuals with no myocardial infarction (non‐MI) in Iraq considering demographic and risk factors utilizing chi‐square tests and univariate logistic regression analysis.

Variable	Non‐MI patients (*N* = 200) Anti*-Toxoplasma* IgG antibody					
	Positive no. (%)	Negative no. (%)	^∗^ *p* value	Odds ratio	95% CI	*p* value
*Age*						
< 30 yrs	1 (20.0)	4 (80.0)				
30–60 yrs	88 (68.2)	41 (53.1)	0.012^∗^	2.76	(1.265–5.431)	*p* ≤ 0.05^∗^
60 < yrs	30 (54.5)	36 (54.5)				

*Gender*						
Male	56 (38.8)	88 (61.2)				
Female	36 (64.2)	20 (35.8)	0.000^∗^	3.42	2.31–5.79	*p* ≤ 0.001^∗^

*Residence*						
Urban	61 (51.7)	57 (48.3)	0.124	1.43	0.76–2.43	0.273
Rural	50 (60.1)	32 (39.9)				

*Agriculture activity*						
No	61 (45.2)	74 (54.8)				
Yes	31 (47.6)	34 (52.4)	0.557	1.33	(0.706–2.228)	0.361

*Consumption of raw/uncooked meat*						
No	52 (34.6)	98 (65.4)				
Yes	40 (80.0)	10 (20.0)	0.000^∗^	3.69	1.432–6.244	*p* ≤ 0.001^∗^

^∗^ *p* value *<* 0.05 is a significant difference.

**TABLE 5:** Multivariate logistic regression analysis to predict the impact of independent variables on *Toxoplasma gondii* seropositivity among patients diagnosed with myocardial infarction (MI) and non‐MI participants in Iraq.

Variable	MI group		Non‐MI group	
	Odds ratio (95% CI)	*p* value	Odds ratio (95% CI)	*p* value
Age	2.14 (1.121–4.21)	0.032^∗^	2.32 (1.19–4.561)	*p* ≤ 0.001^∗^
Gender	3.21 (1.521–7.321)	0.002^∗^	2.724 (1.176–6.414)	*p* ≤ 0.05^∗^
Residence	1.22 (0.8.6–1.894)	0.392	1.41 (0.731–2.593)	0.28
Agriculture activity	1.23 (0.716–2.372)	0.170	1.621 (0.763–1.125)	0.262
Consumption of raw/uncooked meat	2.26 (1.37–3.861)	0.003^∗^	2.96 (1.823–6.734)	*p* ≤ 0.001^∗^

^∗^ *p* value *<* 0.05 is a significant difference.

### 3.4. The Serum Level of Cytokines

**FIGURE 1:**
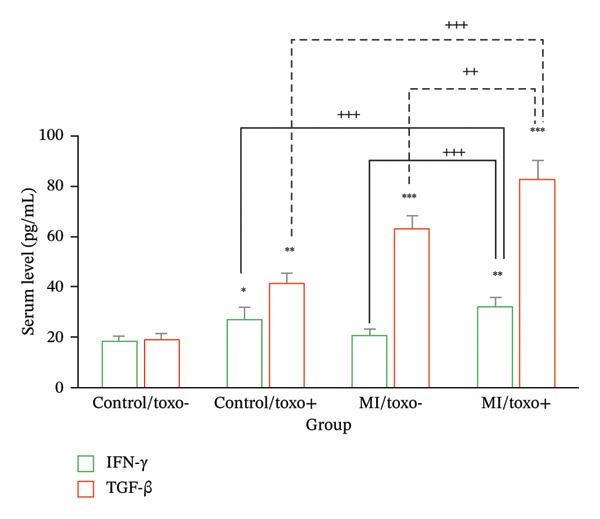
The serum concentrations of transforming growth factor beta (TGF‐β) and interferon‐gamma (IFN‐γ) in patients diagnosed with myocardial infarction (MI) and non‐MI participants in Iraq. This comparison is further stratified based on seropositivity to *Toxoplasma gondii* antibodies (Toxo), utilizing the enzyme‐linked immunosorbent assay (ELISA) methodology. The data are presented as mean values with standard deviations. Statistical significance is indicated as follows: ^∗^ *p* ≤ 0.05; ^∗∗^ *p* ≤ 0.01; ^∗∗∗^ *p* ≤ 0.001, denoting significant differences when compared to the non‐MI patients who were seronegative to toxoplasmosis by a post hoc analysis; ^++^ *p* ≤ 0.01 and ^+++^ *p* ≤ 0.001 indicate a significant difference identified through the post hoc analysis.

The findings of analysis of variance showed considerable differences in the values of the serum concentration of IFN‐γ and TGF‐β in the MI and non‐MI groups (*p* ≤ 0.001) (Figure 1). The analysis concluded that patients with MI and *T. gondii* infection were characterized by a mean concentration of serum IFN‐γ that was 31.15 pg/mL on average, compared with 19.7 pg/mL in patients without *T. gondii* infection. However, this was a meaningful difference (*p* ≤ 0.05) between the two patient groups. Furthermore, it was noted that the values of the serum concentration of the cytokine IFN‐γ, relative to *T. gondii* infection, were greatly elevated even in non‐MI patients at a concentration of 27.0 pg/mL, whereas the control group, i.e., non‐MI patients without *T. gondii* infection, measured 17.4 pg/mL (*p* ≤ 0.05). The values of the serum concentration of the cytokine TGF‐β were analyzed with regard to patients with MI, with comparisons made with regard to *T. gondii* infection status, i.e., with and without infection by the parasite *T. gondii*. The analysis revealed significant differences with regard to non‐MI patients with non‐TG infection, with the mean concentration of 64.55 pg/mL, whereas with *T. gondii*. Furthermore, the serum concentration of TGF‐β in non‐MI/non–*T. gondii* was measured at 18.11 pg/mL, whereas non‐MI participants who were seropositive for *T. gondii* exhibited a TGF‐β level of 41.15 pg/mL (*p* ≤ 0.001). To determine the specific groups responsible for these differences, Tukey’s post hoc analysis was performed. The average levels of IFN‐γ and TGF‐β in MI patients with *T. gondii* antibodies were notably elevated compared to the other three groups (*p* ≤ 0.001).

### 3.5. Evaluating the Expression Level of IFN‐γ and TGF‐β Genes

**FIGURE 2:**
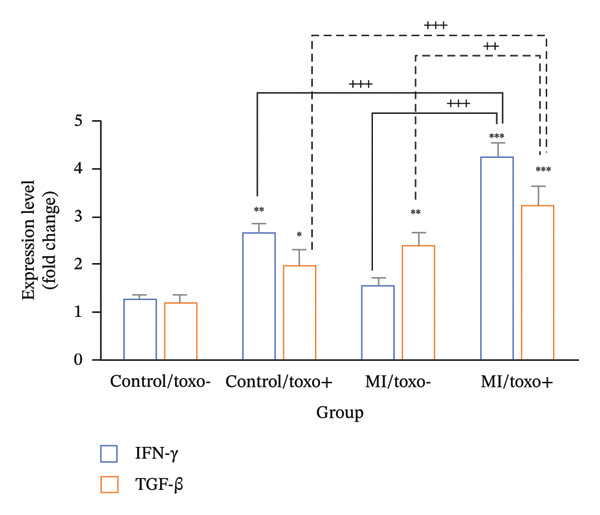
The expression level of transforming growth factor beta (TGF‐β) and interferon‐gamma (IFN‐γ) in patients diagnosed with myocardial infarction (MI) and non‐MI participants in Iraq. This comparison is further stratified based on seropositivity to *Toxoplasma gondii* antibodies (Toxo), utilizing the enzyme‐linked immunosorbent assay (ELISA) methodology. The data are presented as mean values with standard deviations. Statistical significance is indicated as follows: ^∗^ *p* ≤ 0.05; ^∗∗^ *p* ≤ 0.01; ^∗∗∗^ *p* ≤ 0.001, denoting significant differences when compared to the non‐MI patients who were seronegative to toxoplasmosis by a post hoc analysis; ^++^ *p* ≤ 0.01 and ^+++^ *p* ≤ 0.001 indicate a significant difference identified through the post hoc analysis.

A highly significant difference was observed in the gene expression levels of IFN‐γ and TGF‐β between MI and non‐MI groups (*p* ≤ 0.001) (Figure 2). Tukey’s post hoc analysis showed that MI/*T. gondii* antibody‐positive participants had 4.21‐fold and 3.24‐fold higher expression of IFN‐γ and TGF‐β, respectively, compared to the other three groups (*p* ≤ 0.001). Meanwhile, in non‐ MI/*T. gondii* antibody‐positive participants, IFN‐γ and TGF‐β expressions were 2.63 and 2.37 times higher than those in their seronegative non‐MI group (*p* ≤ 0.05).

## 4. Discussion

Over the years, it has been well‐documented that toxoplasmosis can result in various cardiovascular conditions, including myocarditis, pericarditis concomitant with myocarditis, and acute heart failure, among others, in affected individuals [5]. Recent data, however, highlight that cardiovascular diseases continue to represent a leading cause of mortality globally, responsible for approximately 18 million deaths annually, which corresponds to an estimated 32% of all deaths worldwide [14]. This gap is notable given that cardiovascular manifestations associated with *T. gondii* infection are relatively infrequent and are often overshadowed by the predominant central nervous system deterioration observed in infected patients.

The findings of our study indicated that 55.5% and 42.9% of participants in the MI and non‐MI groups were found to have *T. gondii* IgG antibodies. Consistent with these observations, Khademvatan et al. (2020) found a significantly higher rate of anti‐*Toxoplasma* IgG antibodies in 375 Iranian patients with cardiovascular diseases (63.73%) compared to 336 healthy controls (37.64%). Another survey demonstrated that in a Romanian cohort, 164 out of 256 patients with cardiovascular diseases (64.06%) and 138 out of 261 healthy volunteers (52.88%) were seropositive for anti‐*Toxoplasma* IgG antibodies. Furthermore, Alvarado‐Esquivel (2016) found that among 400 patients with heart diseases in Mexico, 45 individuals (13.8%) were positive for these antibodies, whereas only 32 out of 400 healthy participants (8.0%) exhibited seropositivity [15]. An Egyptian study compared 160 individuals with coronary atherosclerosis to 160 without it and found 63.1% and 46.2% had *Toxoplasma* antibodies, respectively [16]. These findings indicate a higher level of anti–*T. gondii* IgG antibodies among patients with coronary atherosclerosis. The present investigation identified that individuals aged 30–40 years, males, and those who consume raw or undercooked meat exhibited an increased risk of *Toxoplasma* infection among patients with MI. Khademvatan et al. (2020) found a significant link between IgG antibodies against *T. gondii* and the consumption of raw or undercooked meat among Iranian patients with cardiovascular diseases [17]. Also, Dragomir et al. [18] conducted a survey on cardiovascular disease patients in Romania that showed a strong connection between IgG antibodies to *T. gondii*, being male and being aged 30–50. These findings suggest that the varying rates of *Toxoplasma* infection in heart disease patients might reflect trends in the general population. The detected variability may be generalized to broader populations. This variability is likely influenced by multiple factors, including the sample size of the respective studies, the sociocultural behaviors of the participants, the demographic characteristics of the population under investigation, the geographical and climatic conditions of the study locations, and the diagnostic techniques employed to detect anti–*T. gondii* antibodies.

Today, it is established that the proinflammatory mediators, for example, IFN‐γ, TGF‐β, TNF‐α, IL‐12, and IL‐1, are critical in initiating and maintaining the immune reactions for controlling *T. gondii* infection [10]. On the contrary, studies demonstrate that these mediators are essential components of the immune response that is crucial in causing cardiovascular conditions through inflammatory mechanisms [9]. Due to the high morbidity and mortality resulting from cardiovascular conditions, monitoring them accurately is of essence. This has steered massive research toward uncovering important biomarkers that will be valuable in the diagnosis and prognosis. The key interleukins involved in causing cardiovascular conditions should be studied. It consolidates knowledge regarding their interaction with the condition and lets everyone know the significant role played by these interleukins in the process [19].

IFN‐γ, classified exclusively within type II interferons, is produced by T lymphocytes and macrophages [20]. It has been shown to induce the production of various cytokines and exert multiple effects throughout the atherogenic process. IFN‐γ activates several signaling pathways resulting in increased oxidative stress, stimulation of smooth muscle cell proliferation and migration into the intima, induction of platelet‐derived factor production, and ultimately contributing to plaque instability in atherosclerosis [21]. These effects underscore only a fraction of the significant role IFN‐γ plays in the initiation and progression of cardiovascular diseases associated with atherosclerosis. The present study clearly demonstrates a significant association between serum IFN‐γ levels and IFN‐γ gene expression in relation to the *T. gondii* antibody presence among MI patients, compared to MI patients without *T. gondii* antibodies and individuals without MI [22].

MI induces immediate cellular necrosis due to ischemic injury. Additionally, myocardial scarring occurs in the form of fibrosis, which contributes to myocardial dysfunction and may lead to subsequent heart failure [23]. Fibrosis results from the excessive accumulation of extracellular matrix components and is primarily mediated by myofibroblasts, which differentiate from normally quiescent cardiac fibroblasts under the influence of TGF‐β and its associated signaling pathways [13]. Consequently, it is imperative to elucidate the role of TGF‐β in the pathogenesis of cardiac fibrosis, cardiomyocyte apoptosis, cardiac hypertrophy, and the progression to heart failure [13]. The present study advocates for the consideration of IFN‐γ and TGF‐β genes as key contributors to the pathogenesis of cardiovascular diseases. The findings underscore the importance of this hypothesis as a foundation for further investigation. However, it is important to note some limitations in the research, such as the relatively small sample size, which could impact how widely the results can be applied to larger and more diverse populations across different regions of Iran. Moreover, it is essential to consider the influence of additional variables, such as cultural, social, and economic factors, which may impact the applicability and interpretation of the findings within the Iranian context.

## 5. Conclusion

The results of the present study demonstrate that patients with MI exhibit elevated anti‐*T. gondii* antibody responses compared to individuals without MI. Furthermore, the data indicate that the cytokines IFN‐γ and TGF‐β play a critical role in the pathogenesis of cardiovascular diseases. These findings suggest a potential modulatory effect of *T. gondii* infection on MI development, mediated through immune system–driven inflammatory processes. Importantly, this study highlights molecular similarities between MI and its associated pathogens, specifically the components of MI and invading agents such as *T. gondii*. The increased levels of IFN‐γ and TGF‐β may serve as valuable biomarkers for investigating the pathogen’s impact on MI incidence.

## Funding

The authors have nothing to report.

## Consent

The authors have nothing to report.

## Conflicts of Interest

The authors declare no conflicts of interest.

## Data Availability

All data generated or analyzed during this study are included in this published article.
